# Adiponectin and Leptin Exert Antagonizing Effects on HUVEC Tube Formation and Migration Modulating the Expression of CXCL1, VEGF, MMP-2 and MMP-9

**DOI:** 10.3390/ijms22147516

**Published:** 2021-07-13

**Authors:** Ersilia Nigro, Marta Mallardo, Rita Polito, Filippo Scialò, Andrea Bianco, Aurora Daniele

**Affiliations:** 1 Department of Environmental, Biological and Pharmaceutical Sciences and Technologies, University of Campania “Luigi Vanvitelli”, 81100 Caserta, Italy; ersilia.nigro@unicampania.it (E.N.); marta.mallardo@unicampania.it (M.M.); polito@ceinge.unina.it (R.P.); 2 CEINGE-Biotecnologie Avanzate Scarl, Via G. Salvatore 486, 80145 Napoli, Italy; scialo@ceinge.unina.it; 3 Department of Translational Medical Sciences, Hospital Monaldi, University of Campania “Luigi Vanvitelli”, 80131 Naples, Italy; andrea.bianco@unicampania.it; 4 Department of Molecular Medicine and Medical Biotechnology (DMMBM), University of Naples “Federico II”, 80131 Naples, Italy

**Keywords:** AdipoRon, leptin, HUVEC endothelial cells, angiogenesis, cell migration

## Abstract

Adiponectin and leptin are two abundant adipokines with different properties but both described such as potent factors regulating angiogenesis. AdipoRon is a small-molecule that, binding to AdipoRs receptors, acts as an adiponectin agonist. Here, we investigated the effects of AdipoRon and leptin on viability, migration and tube formation on a human in vitro model, the human umbilical vein endothelial cells (HUVEC) focusing on the expression of the main endothelial angiogenic factors: hypoxia-inducible factor 1-alpha (HIF-1α), C-X-C motif chemokine ligand 1 (CXCL1), vascular endothelial growth factor A (VEGF-A), matrix metallopeptidase 2 (MMP-2) and matrix metallopeptidase 9 (MMP-9). Treatments with VEGF-A were used as positive control. Our data revealed that, at 24 h treatment, proliferation of HUVEC endothelial cells was not influenced by AdipoRon or leptin administration; after 48 h longer exposure time, the viability was negatively influenced by AdipoRon while leptin treatment and the combination of AdipoRon+leptin produced no effects. In addition, AdipoRon induced a significant increase in complete tubular structures together with induction of cell migration while, on the contrary, leptin did not induce tube formation and inhibited cell migration; interestingly, the co-treatment with both AdipoRon and leptin determined a significant decrease of the tubular structures and cell migration indicating that leptin antagonizes AdipoRon effects. Finally, we found that the effects induced by AdipoRon administration are accompanied by an increase in the expression of CXCL1, VEGF-A, MMP-2 and MMP-9. In conclusion, our data sustain the active role of adiponectin and leptin in linking adipose tissue with the vascular endothelium encouraging the further deepening of the role of adipokines in new vessel’s formation, to candidate them as therapeutic targets.

## 1. Introduction

The vascular system is a complex biological system that plays a crucial role in various physio-pathological conditions such as tumor development, cardiovascular diseases (CVD) and atherosclerosis [1]. This latter is at the basis of majority of the vascular events, some of these that might lead to mortality. Furthermore, endothelial dysfunction, inflammatory response and neovascularization are important factors affecting vascular remodeling and atherosclerosis progression [2].

Obesity has been recognized as the major risk factor for the metabolic diseases included CVD and atherosclerosis, all characterized by chronic low-grade inflammatory state, hypoxia state and adipose tissue dysregulation [3]. This latter tissue produces different adipokines involved in energy metabolism, immune processes and angiogenesis [4]. Among the others, adiponectin and leptin have recently described as potential factors regulating angiogenesis [5].

Adiponectin is a 30 kDa serum protein, encoded by the *ADIPOQ* gene on chromosome 3. It circulates at high concentrations (3–30 μg/mL) [6] that, in addition to its metabolic actions (insulin sensitizing), possesses anti-atherogenic and anti-inflammatory properties [7]. Recently, hypoadiponectinemia was evidenced in endothelial dysfunction and vessel wall diseases [8,9]. Furthermore, most of the published studies have shown that adiponectin exerts significant effects on vessel angiogenesis even if with contrasting results [10,11]. AdipoRon is an active synthetic adiponectin receptor agonist developed by Okada-Iwabu et al. that binds to AdipoR1 and AdipoR2 and is reported as a promising agent for treating different diseases [12]. Both canonical adiponectin receptors have been found expressed in human umbilical vein endothelial cells (HUVEC) [13]. T-cadherin seems to be essential for mediating the vascular actions of adiponectin [14]. In addition, T-cadherin, a member of the cadherin family of cell adhesion molecules, is widely expressed in the cardiovascular system and seems to participate to the regulation of the angiogenesis process [15,16].

Leptin is a 16 kDa protein involved in regulation of food intake and energy homeostasis as well as of inflammatory and immune reactions [17]. Leptin has been shown to exert opposite functions to adiponectin in several cellular processes and also in the regulation of atherogenic and thrombotic effects [18,19]. Few studies reported angiogenic effects of leptin [20,21,22] with inconclusive results. Leptin, through the leptin receptor ObRb, may potentiate vascular endothelial growth factor (VEGF)-mediated angiogenesis dose-dependently through the increase of endothelial cell VEGF secretion [23,24,25,26]. In addition, it has been proved that induction of FGF2 as well as of the matrix metalloproteinase-2 (MMP-2) and MMP-9 signaling pathways are necessary for leptin angiogenesis [25,27]. However, it is to notice that most of the data suggest a role for leptin in mediating cancer aberrant angiogenesis [28].

In this scenario and considering that, to our knowledge, no data are available about the angiogenic effects of a combined treatment with adiponectin and leptin on HUVEC endothelial cells and if and how the two adipokines interact, we undertook this study to investigate the effects following the treatment of primary HUVEC endothelial cells with AdipoRon and/or leptin on proliferation, angiogenesis and wound healing. In addition, we explored whether the treatments with AdipoRon or leptin are associated with changes in the expression of endothelial angiogenic factors, and specifically of hypoxia-inducible factor 1-alpha (HIF-1α), C-X-C motif chemokine ligand 1 (CXCL1), VEGF-A, MMP-2 and MMP-9. Additionally, since literature data reported that leptin seems to exert opposite adiponectin actions, we evaluated also the combined effect of these adipokines on HUVEC endothelial cells. Treatments with VEGF-A, a key mediator of angiogenesis, were used as positive controls.

## 2. Results

### 2.1. AdipoRon Reduces Cell Viability of HUVEC Endothelial Cells

**Figure 1:**
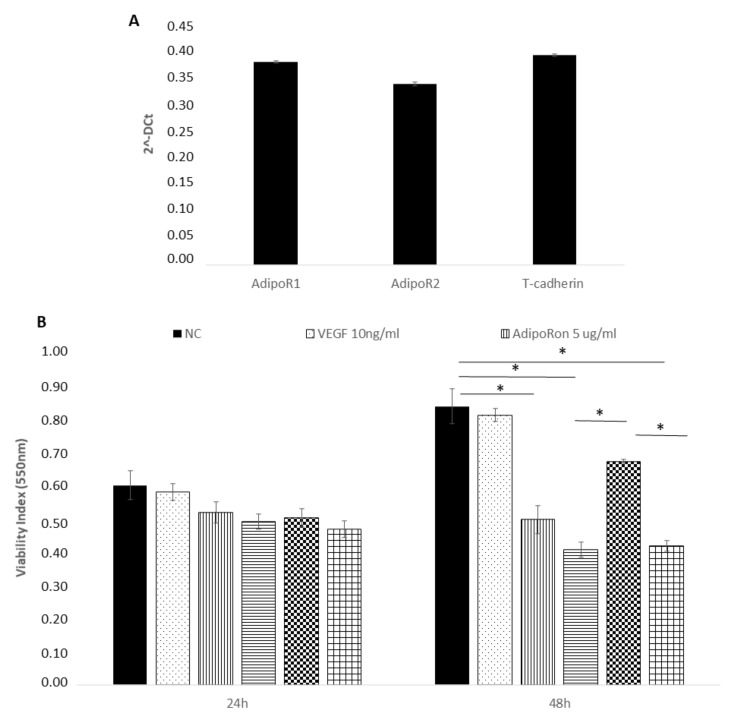
AdipoRon and leptin exert antagonizing effects on HUVEC endothelial cells viability. Adiponectin receptors are expressed on HUVEC endothelial cells as demonstrated by real time PCR (**A**). HUVEC cells were treated with VEGF (10 ng/mL), AdipoRon (5 or 10 μg/mL), leptin (125 ng/mL) and a combination of AdipoRon+leptin (5 μg/mL + 125 ng/mL, respectively) for 24 and 48 h and viability was measured (**B**). Data are reported as mean ± standard deviation (SD) of three independent experiments were reported. * *p* < 0.05. Untreated cells were considered as the negative control.

We first confirmed the presence of adiponectin receptors on HUVEC endothelial cells by real time PCR and found the expression of AdipoR1, AdipoR2 and T-cadherin (Figure 1, panel A). The expression of the Leptin receptor, OB-R, has been previously demonstrated [29,30,31].

Successively, we explored the influence of AdipoRon (5 or 10 μg/mL), Leptin (125 ng/mL) and/or combined treatment (5 μg/mL + 125 ng/mL, respectively) on HUVEC endothelial cells viability. The results revealed that proliferation of HUVEC endothelial cells was not influenced by any of the two molecules at 24 h treatment (Figure 1, panel B). Therefore, these experimental conditions were applied in the next experiments. After a longer exposure time (48 h), the viability, with a dose-dependent course, was negatively influenced by AdipoRon and by the combination of AdipoRon and leptin, while leptin alone produced no effects. Treatment with VEGF-A (10 ng/mL) did not affect cell viability. Untreated cells were considered as the negative control.

### 2.2. AdipoRon and Leptin Exert Antagonizing Effects on HUVEC Tube Formation

**Figure 2:**
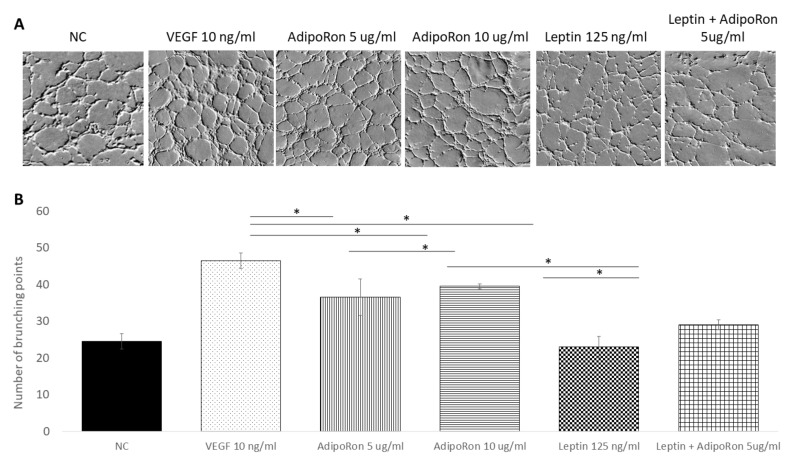
AdipoRon and leptin exert antagonizing effects on tube formation. HUVEC endothelial cells were treated with AdipoRon (5 or 10 μg/mL), Leptin (125 ng/mL) and a combination of AdipoRon+leptin (5 μg/mL and 125 ng/mL, respectively) for 4 h. Panel (**A**) shows representative images of tube formation; panel (**B**) show the mean quantitation of complete tubular structures of three independent experiments. Both doses of AdipoRon induced relevant angiogenesis compared to untreated cells. Leptin does not induce angiogenesis; in the combinational treatment, Leptin antagonizes AdipoRon effects. Untreated cells were considered as the negative control; cells treated with VEGF-A (10 ng/mL) was considered as a positive control. Data are reported as mean ± standard deviation (SD) of three independent experiments. * *p* < 0.05.

In order to investigate the role of AdipoRon (5 or 10 μg/mL), leptin (125 ng/mL) and/or combined treatment (5 μg/mL + 125 ng/mL, respectively) on HUVEC cell angiogenesis, we used the tube formation assay after 4 h of each treatment. As expected, the HUVEC cells growth in the basal medium (NC) had low ability to form tubes, while, when supplemented with VEGF-A (10 ng/mL), had high ability to form complete tubular structure (see Figure 2). Interestingly, as shown in Figure 2, a significant increase in the mean number of complete tubular structures was observed when HUVEC endothelial cells were grown in presence of AdipoRon; indeed, both 5 and 10 μg/mL are effective but the highest dose induced a tube formation comparable to that induced by VEGF-A. On the other hand, supplementation with Leptin (125 ng/mL) did not induce angiogenic tube formation. Interestingly, when cells were treated with a combination of AdipoRon and Leptin (5 μg/mL + 125 ng/mL), no effects on the formation of angiogenic tubes was found, indicating that leptin antagonizes AdipoRon angiogenic effects.

### 2.3. AdipoRon and Leptin Exert Antagonizing Effects on HUVEC Cell Migration

**Figure 3:**
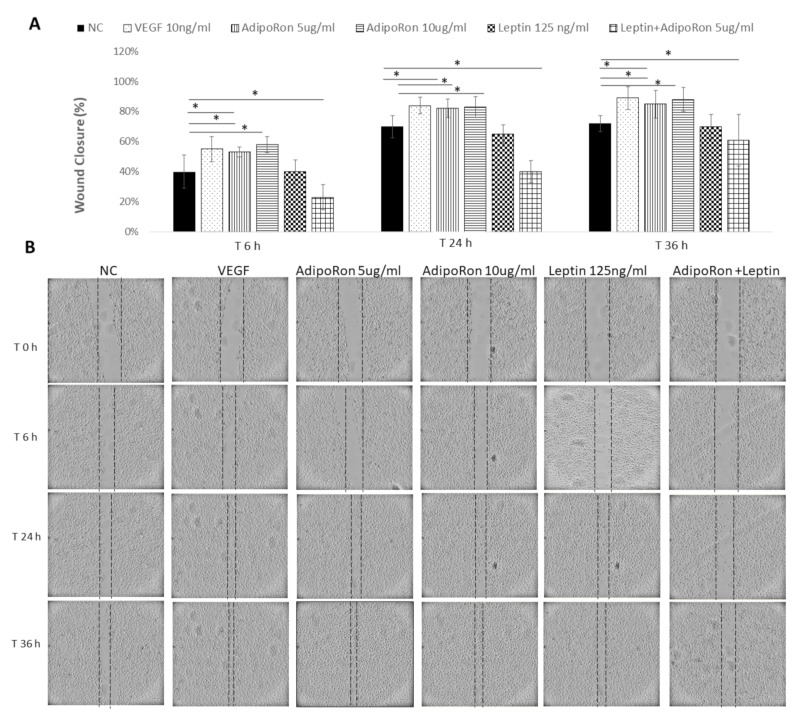
AdipoRon and Leptin exert antagonizing effects on cell migration. HUVEC endothelial cells were subject to a scraped wound and, next, treated with two different doses of AdipoRon (5 or 10 μg/mL) or Leptin (125 ng/mL) alone or in combination. Cells were photographed immediately following the scratch (0 h), and after 6, 24 and 36 h. Untreated cells were considered as the negative control; cells treated with VEGF-A (10 ng/mL) was considered as a positive control. (**A**) Wound closure was measured by calculating pixel densities in the wound area and expressed as percentage of wound closure ± SD. * *p* < 0.05. (**B**) Representative figures are shown from one of two independent experiments.

Considering that endothelial cell migration is an essential component of angiogenesis, and taking into account that adiponectin and leptin might influence the motility and migration, we evaluated the effects of both adipokines on HUVEC endothelial cells migratory activity. To this aim, cells were subjected to a scraped wound and treated with two different doses of AdipoRon (5 or 10 μg/mL) or Leptin (125 ng/mL), alone or in combination at different time points: 6, 24 and 36 h. VEGF-A (10 ng/mL) was used as a positive control, while untreated cells were negative control. AdipoRon significantly induced the migration of the cells in a dose-dependent manner in comparison to untreated cells. On the contrary, the treatment with Leptin inhibited their migration ability. Interestingly, when cells were treated with a combination of AdipoRon and Leptin (5 μg/mL + 125 ng/mL), no effects on the induction of migration was registered, indicating that Leptin antagonizes AdipoRon effects (see Figure 3).

### 2.4. AdipoRon and Leptin Exert Antagonizing Effects on HIF-1α, CXCL1, VEGF-A, MMP-2 and MMP-9 Expression in HUVEC Endothelial Cells

**Figure 4:**
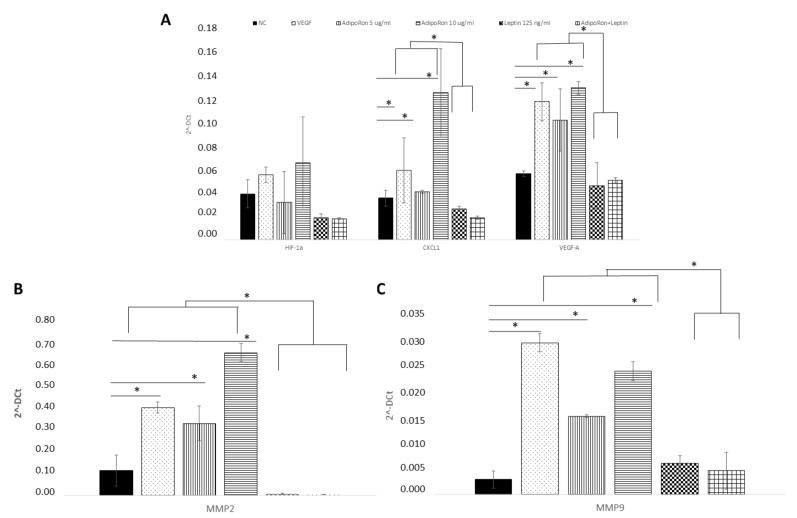
AdipoRon and leptin exert antagonizing effects on HIF-1α, CXCL1, VEGF-A, (**A**) MMP-2 (**B**) and MMP-9 (**C**) expression. HUVEC endothelial cells were treated with AdipoRon (5 or 10 μg/mL), Leptin (125 ng/mL) and a combination of AdipoRon+leptin (5 μg/mL and 125 ng/mL, respectively) for 24 h. Both doses of AdipoRon had effect in increasing the expression of CXCL1, VEGF, MMP2 and MMP9 but not that of H1F1α. Leptin had no effects as well as the combination of AdipoRon+leptin. Untreated cells were used as negative control (NC); VEGF (10 ng/mL) was used as a positive control. mRNA expression levels were determined by RT-PCR in *n * =  3 experiments. Data were standardized employing GAPDH and later quantified by 2^−^ΔΔCt method. * *p* < 0.05.

HUVEC endothelial cells were treated for 24 h with AdipoRon (5 or 10 μg), leptin (125 ng/mL) or in combination (5 μg/mL + 125 ng/mL) to evaluate the potential expression changes in HIF-1α, CXCL1, VEGF-A, MMP-2 and MMP-9 peptides at both mRNA and protein levels. As shown in Figure 4, Quantitative Real-time PCR showed that AdipoRon treatment promotes significant elevation in mRNA levels of CXCL1, VEGF-A, MMP-2 and MMP-9 but not that of HIF1α compared to control, while on the contrary leptin did not affect their expression. Interestingly, when cells were treated with a combination of AdipoRon and leptin, no induction of CXCL1, VEGF-A, MMP-2 and MMP-9 was detected, suggesting that leptin antagonizes AdipoRon effects. VEGF-A (10 ng/mL), used as positive control, induced an increase in the expression levels of both MMP-2 and MMP-9. Untreated cells were considered as the negative control.

**Figure 5:**
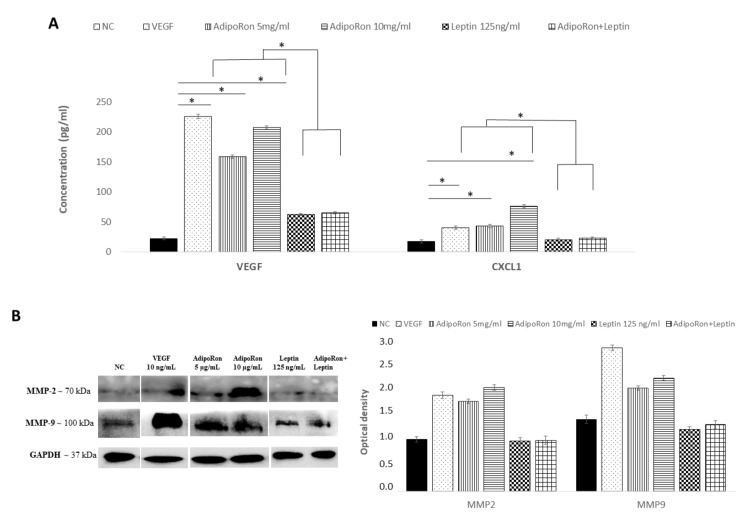
AdipoRon and leptin exert antagonizing effects on HIF-1α, CXCL1, VEGF-A, MMP-2 and MMP-9 protein levels. (**A**) Elisa assay of CXCL1 and VEGF-A; (**B**) representative images of immunoblotting of total MMP-2 and MMP-9 proteins and their quantitation. HUVEC endothelial cells were treated with AdipoRon (5 or 10 μg/mL), Leptin (125 ng/mL) and a combination of AdipoRon+leptin (5 μg/mL and 125 ng/mL, respectively) for 24 h. Untreated cells were used as negative control (NC); VEGF (10 ng/mL) was used as a positive control. Data are reported as mean ± Standard Deviation (SD) of three independent experiments were reported. * *p* < 0.05.

The analysis of the protein levels expression confirmed the results obtained at the mRNA level. As shown in Figure 5, CXCL1 and VEGF-A proteins were induced by AdipoRon treatment and not modulated by leptin treatment or the combination of leptin+AdipoRon (Figure 5, panel A). Similarly, MMP-2 and MMP-9 expression, detected through western blotting analysis, was promoted by AdipoRon treatment but not by leptin treatment or the combination of leptin+AdipoRon (Figure 5, panel B).

## 3. Discussion

To date, the biological role and mechanisms by which adiponectin and leptin act on the angiogenesis processes are still not yet fully understood. Then, and taking into account the unstable and dysfunctional endothelium linked to metabolic diseases, we tested the effects of AdipoRon, an adiponectin agonist, and of Leptin (alone or in combination) on primary HUVEC endothelial cells as an in vitro model of endothelium [32] showing that AdipoRon administration reduces cell viability and promotes angiogenesis and migration capacity through the expression of CXCL1, VEGF, MMP2 and MMP9; on the contrary, Leptin does not affect cell viability and angiogenesis but, interestingly, in the treatments performed in combined manner, leptin antagonizes AdipoRon effects.

Recently, it has become increasingly evident that a dysregulated angiogenesis contributes to the pathogenesis of several diseases, probably due to the complex interplay of multiple molecular signals required to build novel and functional vessels. In particular, it seems to be very promising to deep the potential endocrine role of adipose tissue in the angiogenesis processes [33,34]. Among the adipokines, hormones that actively participate in metabolic and/or inflammatory processes, adiponectin and Leptin are the most abundantly secreted and represent a critical link among nutritional status, metabolism and immunity. In addition, in vitro studies have demonstrated that both adiponectin and leptin have a specific role on endothelial cell proliferation and on new vessels formation [35]. However, the molecular mechanisms that adiponectin and Leptin exert when used alone or in combination on HUVEC angiogenesis are still not yet fully understood.

In this scenario, we investigated AdipoRon effects on HUVEC endothelial cells proliferation observing that it decreases cell proliferation becoming toxic after 48 h of incubation. In line with our data, Dubois et al. reported that adiponectin can inhibit HUVEC endothelial cell growth [36] while, on the contrary, Yan Lu et al., in the same cellular model, showed that adiponectin does not affect cell viability [37]. In addition, our data show that Leptin does not exert any relevant effect in term of cell viability. Dubois et al. found the same trend with no effects of leptin treatment while Park et al. showed that Leptin induces proliferation of HUVEC endothelial cells. However, in accordance with our data, Alvarez et al. demonstrated that leptin does not influence the human microvascular endothelial cell line (HMEC-1) proliferation [38]. No data are available in this cell model about the effects of stimulation with both adipokines on cell viability.

Regarding angiogenesis, our data support that AdipoRon stimulates HUVEC endothelial cells to form complete tube structures; in accordance with our evidence, Malih et al. demonstrated that preconditioning of mesenchymal stem cells with AdipoRon enhances cell survival, angiogenesis and migration via enhancing of HIF-1α, C-X-C chemokine receptor type 4 (CXCR4), C–C chemokine receptor type 2 (CCR2), VEGF, MMP-2 and MMP-9 factors. In addition, we found that angiogenesis is accompanied by an induction of cell migration. Ouchi and colleagues demonstrated that Adiponectin functions as a chemoattractant in migration of HUVEC endothelial cells stimulating the differentiation into capillary-like structures. On the contrary, it is important to notice that an opposite effect, i.e., of angiogenesis inhibition, has been demonstrated in pancreatic, and breast tumor cells [39,40] suggesting that AdipoRon might exert specific effects according to the different cell types used. In particular, in the context of tumor cells, adiponectin and AdipoRon repress the angiogenesis, which represent a crucial step in the malignancy’s development. On the other hand, although it is not clear whether leptin could exert paracrine effects on endothelial cells in vivo, this adipokine has been defined as a potent angiogenic factor mainly involved in the angiogenesis related to tumorigenesis and metastasis in vitro [41]. However, despite several strong evidences reporting pro-angiogenic effects, a recent study has reported that in two clinical trials, NCT00140205 and NCT00130117, Leptin does not regulate circulating angiogenesis related factors [42].

Considering that endothelial cell migration is an essential component of angiogenesis, we evaluated the effects of both leptin and AdipoRon on HUVEC endothelial cells migratory activity finding that the first induces migration while the second one does have any effect. Literature data show that adiponectin may have different effects on endothelial cell migration, depending on cell conditions. Bråkenhielm et al. reported that adiponectin is unable to modify migration ability in HMEC-1 cells [11]. In contrast, adiponectin has also been reported to reduce VEGF-induced endothelial cell migration [43]. Furthermore, in addition with our data, several studies have suggested that adiponectin may act as a chemotactic agent of endothelial cells [10]. Similarly, to adiponectin leptin also appears be an important regulator of cell motility. Indeed, although at low doses leptin is unable to modify the migration in response to a scratch wound, at a high dose it is able to reduce the migration of endothelial progenitor cells [44], However, on the contrary, A’lvarez et al. reported that leptin can act as a chemoattractant factor in endothelial cells [38], while Bråkenhielm et al. reported no effects on HMEC-1 cells [11].

The molecular mechanisms and the mediators involved in angiogenesis by AdipoRon have not still been completely elucidated; however, our data suggest that AdipoRon might possibly act through CXCL1, MMP-9 and MMP-2, -9 expression. In line with this observation, Yung-Taek Ouh et al. demonstrated that adiponectin induces angiogenesis via CXCL1 overexpression in ovarian cancer cells [45]. The inverse relationship between adiponectin’s angiogenetic action and the regulation of MMPs expression has also been suggested in Endometrial cells, where adiponectin reduced angiogenesis in association with a decreased activity of MMP-2 and MMP-9, and an increased TIMP-3 mRNA [46]. As we demonstrated in our cell model, the induction of MMPs by adiponectin results in a facilitated increased migration in a model of cardiac fibroblasts [47]. Dadson and colleagues also found that, consequent to MMPs induction, adiponectin is able to induce a reorganization of the extracellular matrix [46]. To our knowledge few reports analyzed adiponectin effects on MMPs expression specifically in HUVEC endothelial cells; Lee et al. demonstrated that the adipokine significantly stimulates the production of VEGF, MMP-1 and MMP-13 in osteoblasts but not in endothelial cells [47].

It is interesting to notice that leptin did not induce any induction in MMPs expression and that it counteracted AdipoRon effects in the co-treatment condition, strongly suggest that the two cytokines have opposite effects on angiogenesis of HUVEC endothelial cells and that, at least in part, the process is related to the regulation of MMPs proteins. To our knowledge no data are available about the relationship between the combined stimulation with both adipokines and MMPS levels, but Park and colleagues demonstrated, contrary to our evidence, that leptin induces the elevation of MMP-2, MMP-9, TIMP-1 and TIMP-2 expression in a dose-dependent manner in HUVEC cells [25].

## 4. Materials and Methods

### 4.1. Chemicals

AdipoRon and Leptin was purchased from Sigma-Aldrich (Cat. N. 924416-43-3, Cat. N. 181030-10-4, respectively; St. Louis, MO, USA), VEGF-A was purchased from Applied Biological Materials Inc. (abm) (Cat. N. Z102117; Richmond, BC, Canada).

### 4.2. HUVEC Cell Culture

Primary human umbilical vein endothelial cells (HUVEC) were purchased from Gibco (Cat. N. A1460901; Thermo Fisher Scientific, Waltham, MA, USA). The cells were cultured in 5% CO2 at 37 °C in Gibco^®^ Medium 200 basal media supplemented with Gibco^®^ Large Vessel Endothelial Supplement (LVES) (Cat. N. A1460901; Thermo Fisher Scientific), 50×. All assays were conducted using low cell passage cells (2–5 passages).

### 4.3. HUVEC Viability by XTT Assay

HUVECs were trypsinised and seeded at 104 cells/well in 96- well plates. After 24 h, complete medium was removed and samples were incubated with different concentrations of AdipoRon (5 or 10 μg/mL), Leptin (125 ng/mL), AdipoRon+leptin (5 μg/mL and 125 ng/mL, respectively). Cells receiving DMSO (0.1%) served as vehicle controls, and were equivalent to no treatment. Cells treated with VEGF-A (10 ng/mL) were considered as positive control. After 24 h and 48 h cell proliferation was assessed by XTT for 4 h. The spectrophotometrically absorbance of each well was measured by a Multilabel counter (Perkin Elmer, Singapore). The wavelength used to measure absorbance of the formazan product was 490 nm and the reference wavelength was 690 nm. Cell viability data were expressed as average ± standard deviation (SD) of three independent experiments.

### 4.4. Wound Healing Assay

Cells were seeded in a 6-well plate in complete culture media and grown to confluence. The day after, cells were treated with 4μg/mL of mitomycin (Cat. N. 50-07-7; Sigma-Aldrich, St. Louis, MO, USA) for 2 h to inhibit cell proliferation and then a wound was inflicted using a tip as previously reported [48]. After washing with phosphate-buffered saline (PBS), cells were incubated with AdipoRon (5 or 10 μg/mL), leptin (125 ng/mL) or their combination (5 μg/mLand 125 ng/mL, respectively) and compared to untreated cells. Cells treated with VEGF-A (10 ng/mL) were considered as positive control. The same positions along the scratch wound were observed and photographed at different time points using an inverted-phase-contrast microscope (Nikon microscope TS100 fluorescence and video camera). The rate of wound closure was calculated with Cell Software (Olympus Biosystem Gmb, Kingston upon Thames, UK) and expressed as percentage of the closure.

### 4.5. Tube Formation Assay on HUVEC

The effect of AdipoRon and/or Leptin on HUVEC differentiation was examined by in vitro Gibco^®^ Angiogenesis Starter Kit (Cat. N. A1460901; Thermo Fisher Scientific). Confluent HUVECs were harvested and diluted (96104 cells) in medium containing AdipoRon (5 or 10 μg/mL) Leptin (125 ng/mL), AdipoRon+leptin (5 μg/mL and 125 ng/mL, respectively); cells receiving DMSO (0.1%) served as vehicle controls and were equivalent to no treatment. Cells treated with VEGF-A (10 ng/mL) were considered as positive control. Cells were then seeded on coated 24-well plates in triplicate at 37 °C for 4 h. Cells receiving DMSO (0.1%) served as vehicle controls, and were equivalent to no treatment. The network-like structures were examined under an inverted microscope (objective, ×4). The tube-like structures were defined as endothelial cord formations that were connected at both ends. The number of branching points were quantified by counting five random fields/well under a microscope as previously reported [49]. Each experiment was repeated five times.

### 4.6. RNA Extraction and Quantitative Real Time-PCR (q-RT-PCR)

Total RNA was isolated from HUVEC cells using TRIzol (Cat. N. 15596026; Invitrogen, Carlsbad, CA, USA). Real-time quantitative PCR was carried out for 40 cycles at a melting temperature of 95 °C for 15 sec and an annealing temperature of 60 °C for 1 min. A dissociation curve was analyzed for each PCR experiment to assess primer–dimer formation or contamination. Relative mRNA level quantifications of target genes were determined using the cycle threshold method with GAPDH as housekeeping gene, and the data were reported as the expression relative to the housekeeping gene. The sequence of primers for AdipoR1, AdipoR2, HIF1a, CXCL1, VEGF-A, MMP2, MMP9 and GAPDH are reported in Table 1. The experiments were performed two times in triplicate.

### 4.7. Western Blotting Assay

Typically, 15 to 30 μg of total cellular protein were loaded in polyacrylamide gel (Bio Rad Laboratories) and separated by SDS-PAGE. Thereafter, proteins were transferred onto nitrocellulose membrane (Sigma-Aldrich) using Mini Trans-Blot BioRad (Bio Rad Laboratories). Before to be incubated overnight at 4 °C with specific primary antibodies (MMP-2 and mMP-9, Invitrogen, 436000; MA5-15886 Waltham, MA, USA), membranes were blocked in no-fat milk (5% *w*/*v*) for 1 h. The day after, Horseradish Peroxidase (HRP) goat anti-rabbit or anti-mouse antibodies were added to the membranes for 1 h at room temperature. TBS Tween-20 (Thermo Fisher Scientific) was used to wash three times the membranes before and after each incubation procedure. Enhanced ChemiLuminescence (ECL) (Euroclone) was employed to detect HRP secondary antibodies signal. To conclude, protein bands were detected with Chemi Doc XRS (Bio-Rad, Hercules, CA, USA).

### 4.8. ELISA Assay

The release of VEGF-A and CXCL1 from HUVEC cells was quantitatively examined from the respective medium samples using the human VEGF-A and CXCL-1 ELISA system (Invitrogen, BMS277-2; BMS2122 Waltham, MA, USA). Samples and standards were performed in duplicate, and each group contains three independent samples.

In brief, the pre-coated microwells from the microplate were washed twice with approximately 400 μL wash buffer per well. After washing, the pre-diluted media (1:2 dilution) were added to the wells. Plates were incubated for 2 h at room temperature. After an additional wash with PBST, 100 μL of biotin-conjugated anti-human primary polyclonal antibody (1:100) was added to the wells and incubated for 1 h at room temperature. After washing with PBST, 100 μL of horseradish peroxidase-labeled streptavidin (1:100) was added to the wells and incubated for 1 h at room temperature. After washing with PBST, color was developed in 3,3′,5,5′-tetramethylbenzidine liquid substrate for about 30 min at room temperature and then stopped with the stop solution. The plate was analyzed with an enzyme immunoassay plate reader at a wavelength of 450 nm.

### 4.9. Statistical Analysis

Two-way mixed ANOVA was performed, followed by Bonferroni post hoc test was carried out, using the StatView software. Data were expressed as mean ± standard deviation (SD) from individual experiments. Differences were considered as significant at <0.05.

## 5. Conclusions

Our data sustain the cross-link between adipose tissue and endothelium through the secretion of adiponectin and leptin in health homeostasis. Furthermore, our findings encourage the deepening the role of adiponectin and leptin in the new vessel’s formation process. In particular, adiponectin might represent a possible therapeutic target in regulating angiogenesis; the use of adiponectin receptor agonists/antagonists represent plausible field of research useful to achieve the control of angiogenesis.

## Figures and Tables

**Table 1:** Primers’ sequence and melting temperature melting of primers used in q-RT-PCR.

Gene	Primer Sequence	Melting Temperature (°C)
GAPDH fw	5′-CAT GGC CTT CCG TGT TCC TA-3′	59.3 °C
GAPDH rev	5′-CCT GCT TCA CCA CCT TCT TGA T-3′	60.3 °C
MMP-2 fw	5′-TGGCAAGTACGGCTTCTGTC-3′	59.3 °C
MMP-2 rev	5′-TTCTTGTCGCGGTCGTAGTC-3′	60.3 °C
MMP-9 fw	5′-TGCGCTACCACCTCGAACTT-3′	59.3 °C
MMP-9 rev	5′-GATGCCATTGACGTCGTCCT-3′	75.6 °C
VEGF-A fw	5′-CGGCGAAGAGAAGAGACACA-3′	59.3 °C
VEGF-A rev	5′-GGAGGAAGGTC- AACCACTCA-3′	59.3 °C
CXCL1 fw	5′-GCGCCCAAACCGAAGTCATA-3′	59.3 °C
CXCL1 rev	5′-ATGGGGGATGCAGGATTGAG-3′	59.3 °C
HIF-1A fw	5′- GAAAGCGCAAGTCTTCAAAG-3′	55.3 °C
HIF-1A rev	5′-TGGGTAGGAGATGGAGATGC-3′	59.3 °C

## Data Availability

The data presented in this study are available on request from the corresponding author.
